# Co-circulation of Influenza A H5, H7, and H9 Viruses and Co-infected Poultry in Live Bird Markets, Cambodia

**DOI:** 10.3201/eid2402.171360

**Published:** 2018-02

**Authors:** Paul F. Horwood, Srey Viseth Horm, Annika Suttie, Sopheak Thet, Phalla Y, Sareth Rith, San Sorn, Davun Holl, Sothyra Tum, Sowath Ly, Erik A. Karlsson, Arnaud Tarantola, Philippe Dussart

**Affiliations:** Australian Institute of Tropical Health and Medicine, James Cook University, Cairns, Queensland, Australia (P.F. Horwood);; Institute Pasteur, Phnom Penh, Cambodia (P.F. Horwood, S.V. Horm, A. Suttie, S. Thet, P. Y, S. Rith, S. Ly, A. Tarantola, E.A. Karlsson, P. Dussart);; Federation University Australia, Churchill, Victoria (A. Suttie);; National Animal Health and Production Research Institute, Cambodian Ministry of Agriculture, Forestry and Fisheries, Phnom Penh (S. Sorn, D. Holl, S. Tum)

**Keywords:** Influenza, avian influenza virus, H5N1, H7N9, H9N2, viruses, live bird markets, avian, co-infection, co-circulation, poultry, duck, chicken, oropharyngeal, cloacal, swab, carcass wash water, southeast Asia, Phnom Penh, Takeo, Cambodia

## Abstract

Longitudinal surveillance of 2 live bird markets in Cambodia revealed year-round, high co-circulation of H5, H7, and H9 influenza viruses. We detected influenza A viruses in 51.3% of ducks and 39.6% of chickens, and co-infections, mainly by H5 and H9 viruses, in 0.8% of ducks and 4.5% of chickens.

A large variety of avian influenza viruses (AIVs) circulate in live bird markets (LBMs) in countries where highly pathogenic influenza A(H5N1) viruses are endemic (*1*). The low pathogenicity AIVs A(H7N9) and A(H9N2) are also potential threats for global public health, related to the ability of these viruses to cause human infections in people in close contact with infected poultry (*2*). The segmented genomes of influenza A viruses indicate that co-infections can result in progeny with mixed genomes. Therefore, co-circulation of a large diversity of AIVs is a risk for emergence of novel reassortant viruses affecting animals, humans, or both.

Cambodia is a developing country in Southeast Asia that has a population of >15 million, of which 73% are dependent on agriculture for their livelihood (*3*). The country is estimated to have a poultry population of ≈18 million chickens and ≈8 million ducks; most are raised in small backyard flocks (*4*). Poultry are generally transported on trucks and motorbikes into centralized LBMs, where they are slaughtered after being sold to customers (*5*). These LBMs have been established as critical for persistence, amplification, and dissemination of AIVs (*6*).

Surveillance studies in LBMs in Cambodia have revealed some of the highest AIV detection rates in poultry globally (*1**,**7*). As of November 2017, a total of 56 human cases (including 37 deaths) and 49 poultry outbreaks of influenza A(H5N1) have been recorded in Cambodia (*8*–*10*). However, little is known about other AIV subtypes at risk for pandemic emergence, mainly H7 and H9. We investigated the circulation of potentially highly pathogenic AIV subtypes (H5, H7, and H9), that have known public health risks in Cambodia LBMs during 2015.

## The Study

We administered a longitudinal survey in 2 LBMs in the highly populated southeast region of Cambodia during February–December (weeks 7–53), 2015. Market 1 is a large LBM in central Phnom Penh that serves as a hub for poultry commerce in the southeast region. Market 2 is a smaller provincial market in Takeo Province. Weekly, we collected pooled oropharyngeal and cloacal swabs from 4 chickens and 4 ducks, randomly selected, in each LBM. We also collected 50-mL samples of carcass wash water (CWW; large buckets of water that are used to wash freshly slaughtered poultry) weekly from each LBM. We transported samples at 4°C, immediately aliquoted them, and stored them at −80°C until testing.

In the Virology Unit Laboratory of Institut Pasteur, Cambodia, we concentrated viruses in the CWW samples as previously described (*11*). We extracted viral RNA from CWW and swab samples by using the QIAamp Viral RNA Mini Kit (QIAGEN, Valencia, CA, USA), according to the manufacturer’s instructions. We then tested extracts for influenza A (M-gene) and subtypes H5 (primer sets H5a and H5b), N1, H7, and H9 by using quantitative RT-PCR (qRT-PCR) or conventional RT-PCR. The H5, H7, and H9 gene targets were all tested by using 2 separate assays to reduce the chances of false-negative results caused by the presence of single-nucleotide polymorphisms. Except for 1 of the H7 qRT-PCR and the H9 conventional RT-PCR, the assays were sourced from the International Reagent Resource (https://www.internationalreagentresource.org/Home.aspx) (*12**,**13*). For the qRT-PCR assays, cycle threshold values <38 were considered positive.

Overall, we collected 940 samples, including 376 chicken swab samples, 376 duck swab samples, and 188 CWW samples. Testing identified AIV RNA year-round from 39.6% of chickens, 51.3% of ducks, and 93.1% of CWW samples (Table 1). We detected H5 in 16.8% of chickens, 20.5% of ducks, and 76.6% of CWW samples; H7 in 0.5% of chickens, 1.1% of ducks, and 2.1% of CWW samples; and H9 10.9% of chickens, 1.1% of ducks, and 40.4% of CWW samples. Market 1 had a higher prevalence than market 2 of most viruses except H7 viruses, which were detected at similar rates in both markets. The higher rate of virus detection might be a result of the substantially larger size of market 1, which accommodated more diverse supply chains than did market 2.

**Table 1:** Positivity rate for avian influenza viruses in live bird markets, Cambodia, February–December 2015*

Type/subtype	Market 1, Phnom Penh, no. (%)				Market 2, Takeo, no. (%)				Combined, no. (%)		
	Chicken, n = 188	Duck, n = 188	CWW, n = 94		Chicken, n = 188	Duck, n = 188	CWW, n = 94		Chicken, n = 376	Duck, n = 376	CWW, n = 188
Influenza A	89 (47.3)	105 (55.9)	86 (91.5)		60 (31.9)	88 (46.8)	89 (94.7)		149 (39.6)	193 (51.3)	175 (93.1)
H5	41 (21.8)	53 (28.2)	71 (75.5)		22 (11.7)	24 (12.8)	73 (77.7)		63 (16.8)	77 (20.5)	144 (76.6)
N1	24 (12.8)	21 (11.2)	48 (51.1)		8 (4.3)	9 (4.8)	62 (66.0)		32 (8.5)	30 (8.0)	110 (58.5)
H7	1 (0.5)	2 (1.1)	1 (1.1)		1 (0.5)	2 (1.1)	3 (3.2)		2 (0.5)	4 (1.1)	4 (2.1)
H9	28 (14.9)	3 (1.6)	54 (57.4)		13 (6.9)	1 (0.5)	22 (23.4)		41 (10.9)	4 (1.1)	76 (40.4)
Co-infections	15 (8.0)	3 (1.6)	44 (46.8)		2 (1.1)	0	20 (21.2)		17 (4.5)	3 (0.8)	64 (34.0)

*By quantitative reverse transcription PCR. n values indicate number of samples. CWW, carcass wash water.

Co-infections were more frequent among chickens (4.5%) than ducks (0.8%), and high viral loads (determined by aRqT-PCR cycle threshold values <30) of co-infecting viruses were detected in many of the chicken samples (Table 2). Most co-infections detected were H5 and H9 viruses in chickens, particularly from market 1.

**Table 2:** Avian influenza co-infections detected in poultry from live bird markets in Cambodia, February–December 2015

Sample code	Market/ week	Bird	Cycle threshold values by quantitative RT-PCR							H9 RT-PCR¶	Co-infections
			Influenza A†‡	H5a†	H5b†	N1†	H7§	H7†	H9†		
Z-47	M1/W9	C	18.72	18.96	ND	23.57	–	–	20.37	+	H5N1/H9
Z-68	M1/W10	C	19.38	26.94	ND	25.41	–	–	30.82	+	H5N1/H9
Z-104	M1/W12	D	24.13	32.73	30.74	32.90	–	–	23.39	+	H5N1/H9
Z-138	M2/W13	C	26.02	28.04	25.84	25.37	–	–	29.02	+	H5N1/H9
Z-150	M1/W14	C	34.25	27.01	39.76	–	–	–	34.47	+	H5/H9
Z-167	M1/W15	C	30.81	38.60	36.36	–	–	–	30.84	+	H5/H9
Z-169	M1/W15	C	27.83	37.93	35.08	–	–	–	27.58	+	H5/H9
Z-170	M1/W15	C	22.79	33.52	32.44	34.79	–	–	25.65	+	H5N1/H9
Z-227	M1/W18	C	28.90	35.77	29.81	30.17	–	–	30.60	+	H5N1/H9
Z-228	M1/W18	C	23.93	29.54	23.63	24.60	–	–	29.84	+	H5N1/H9
Z-230#	M1/W18	C	25.61	–	39.71	38.54	–	–	22.65	+	H5N1/H9
Z-267	M1/W20	C	22.70	28.69	26.80	30.18	–	–	27.66	+	H5N1/H9
Z-269#	M1/W20	C	21.04	–	–	–	–	–	27.00	+	H5N1/H9
Z-430	M1/W28	C	23.79	–	–	–	–	24.78	24.98	+	H7/H9
Z-466	M1/W30	D	32.56	40.93	–	–	–	34.72	36.00	+	H7/H9
Z-568#	M1/W35	C	32.40	–	41.34	–	–	–	37.39	+	H5N1/H9
Z-748#	M1/W44	C	21.05	–	35.44	37.75	–	–	27.17	+	H5N1/H9
Z-824	M1/W48	D	19.40	17.20	23.82	25.38	–	–	31.79	–	H5N1/H9
Z-858#	M2/W49	C	18.41	40.23	–	24.02	–	–	27.67	+	H5N1/H9
Z-909#	M1/W52	C	24.14	38.03	39.37	–	–	–	29.88	+	H5/H9

*C, chicken; D, duck; RT-PCR, reverse transcription PCR; +, positive; –, negative. †US Centers for Disease Control and Prevention assays, available at the International Reagent Resource (https://www.internationalreagentresource.org/Home.aspx). ‡All samples with an M gene quantitative RT-PCR cycle threshold value <38 were inoculated into embryonated chicken eggs to confirm avian influenza infection. §Van Borm et al. (*13*). ¶World Health Organization (*12*). #Equivocal H5 or N1 results (defined by cycle threshold value >38) were subsequently confirmed by isolation of influenza A(H5N1) in embryonated chicken eggs (data not shown).

The H5 subtype (37.2% of birds) was detected much more frequently than N1 (16.5%) by using the subtype-specific qRT-PCR assays. Previous studies have suggested that the sensitivities of M and H5 qRT-PCRs are higher than that for N1 (*1*). Isolation and sequence analysis of samples that were H5 positive and N1 negative revealed no evidence for circulation of non-N1 H5 strains. Similarly, further investigations were undertaken to confirm that none of the H7 viruses were H7N9 or HPAI strains (data not shown). The circulation of Eurasian-lineage LPAI H7 viruses in the region has been established through surveillance in South Korea (*14*).

The peak in AIV and H5N1 circulation occurred during February–April, with a secondary peak during November–December (Figure). Surveillance was not extended into January 2016; however, we assume that the peak period extends from November through April, coinciding with the dry season in Cambodia. Similar seasonality of AIV circulation was observed in a longitudinal LBM study in 2013, in which February was also the month with the highest prevalence of AIV-infected poultry (*1*). Knowledge of this peak period of circulation is needed so that animal and human health authorities can target interventions to reduce AIV spread and human exposure. We detected H9 circulation in chickens year-round, with no discernable seasonality. H7 seasonality could not be determined because of the small number of viruses detected.

**Figure:**
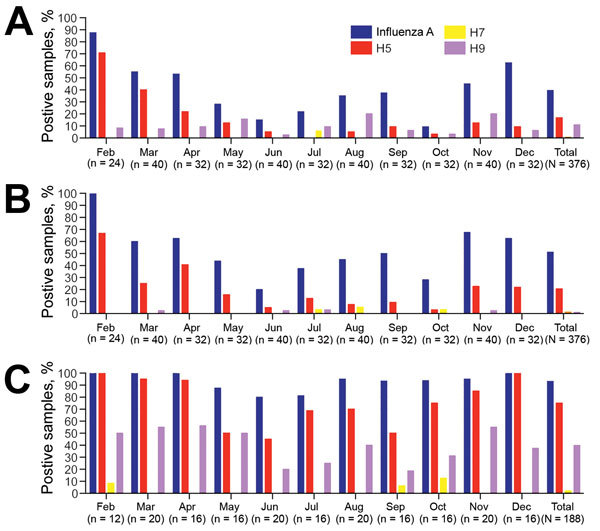
Seasonality of avian influenza subtype circulation in live bird markets in Cambodia during 2015, as detected by quantitative reverse transcription PCR. A) Oropharyngeal and cloacal swabs from chickens; B) oropharyngeal and cloacal swabs from ducks; C) carcass wash water samples from pooled chickens and ducks after euthanization.

## Conclusions

Throughout the study, we detected high levels of AIVs in 2 LBMs in southeastern Cambodia. In 2013, our research team conducted a similar longitudinal AIV surveillance study that included the same 2 LBMs (referred to as M1, Phnom Penh, and M4, Takeo, in the 2013 study [*1*]). In that study, we detected AIVs in 32% of ducks, 18% of chickens, and 75% of CWW samples. This new study found a substantial increase in circulation of AIVs: 51% of ducks, 40% of chickens, and 93% of CWW samples. However, no corresponding increase in cases of H5N1 infection among humans was detected in Cambodia during 2015, possibly related to the replacement of the H5N1 clade 1.1.2 reassortant virus with an H5N1 clade 2.3.2.1c virus in early 2014.

The detection of co-infections in 4.5% of chickens during this study was cause for concern. Reassortment between H5N1 and other AIVs could produce novel viruses that have potential to cause epizootics or pandemic emergence. Reassortment with the internal genes of H9N2 viruses has been linked to the emergence of numerous AIVs that raise public health concern, such as H5N1, H7N9, and H10N8 (*15*). In this study, only H5, H7, and H9 subtypes were screened, because strains of these viruses (particularly H5N1, H7N9, and H9N2) are of leading global health concern. We would expect an even higher detection rate of co-infections if all AIV subtypes were tested.

In summary, we have documented a substantial increase in the prevalence of AIVs in Cambodian LBMs from 2011 (*7*) to 2013 (*1*) to 2015. We also have established that co-infections between AIVs commonly occur in the LBM environment and there is potential for emergence of novel viruses through reassortment. Interventions should be considered to decrease the prevalence of AIVs in LBMs to reduce the risk for emergence of novel viruses.
